# Behavioral and kinematic outcomes of adaptive pauses in VR social cognition training for autistic children

**DOI:** 10.3389/fpsyg.2026.1827283

**Published:** 2026-05-29

**Authors:** Luna Maddalon, Alberto Altozano, Maria Eleonora Minissi, Gary Lovaton Romero, Sara Gámez Martínez, Javier Marín-Morales, Amaia Hervás, Mariano Alcañiz

**Affiliations:** 1 Human-Centered Technology Research Institute (HUMAN-Tech), Polytechnic University of Valencia, Valencia, Spain; 2 Fundació de Docencia Y Recerca Mútua Terrassa (MTA), Grup Salut Mental Infanto Juvenil, Barcelona, Spain

**Keywords:** adaptive pause, autism spectrum disorder, kinematic analysis, personalized intervention, statistical learning, virtual reality

## Abstract

**Background:**

Autism Spectrum Disorder (ASD) is characterized by persistent challenges in social communication and restricted, repetitive behavioral patterns. Virtual Reality (VR) has emerged as a promising tool for social-cognitive rehabilitation, yet optimizing these interventions through personalized pacing remains underexplored. Adaptive Pauses (APs)—brief regulatory breaks designed to regulate arousal and prevent cognitive overload—have emerged as a key mechanism for sustaining engagement and optimizing learning.

**Objective:**

This study expands on a preliminary, validated VR-based social cognition intervention and examines the comparative efficacy of three AP implementation methods: rule-based, Wizard of Oz (human-triggered), automated-random (algorithm-driven), and no-AP.

**Methods:**

Forty-six autistic children with level 1 were randomly assigned to one of the four conditions. Each VR session embedded APs consisting of guided breathing and sensory relaxation. Behavioral and kinematic data were continuously recorded across sessions, alongside pre- and post-intervention assessments of social cognition.

**Results:**

Significant improvements in social-cognitive outcomes were observed from pre- to post-test, replicating earlier findings. Behavioral indices showed a trend toward improvement across sessions, and kinematic analyses indicated increases in acceleration and displacement, suggesting more comfortable environmental conditions. However, no significant differences emerged among the four groups in any outcome measure.

**Conclusions:**

VR-based interventions effectively improved social cognition in autistic children, as measured by ToMTB, with robust session-driven gains in behavioral performance and kinematics, supporting the main result. While AP methods did not produce differential intervention outcomes, the findings establish the feasibility and safety of integrating structured pauses into immersive interventions without impeding progress. However, conclusions regarding the efficacy of APs remain preliminary due to limited statistical power and absence of generalization beyond the experimental context.

## 1 Introduction

Autism Spectrum Disorder (ASD) is a heterogeneous neurodevelopmental condition characterized by persistent impairments in social communication and interaction, alongside restricted and repetitive patterns of behavior (American Psychiatric Association, 2013; Zhao et al., 2026). Central to these challenges are profound deficits in social cognition, specifically in the Theory of Mind (ToM) framework. Individuals struggle with higher-order functions such as mental-state attribution, understanding of false beliefs, and integrating social cues into a coherent narrative (Baron-Cohen, 2001). Consequently, these deficits significantly affect educational, relational, and adaptive functioning across development. Traditional social skills interventions often rely on role-playing or didactic instruction. While beneficial, such approaches may lack the ecological validity necessary for real-world skill generalization. Thus, there is a clinical need for interventions that provide safe, controlled, and repeatable environments in which social-cognitive skills can be systematically practiced. The emergence of immersive Virtual Reality (VR) has provided a groundbreaking pathway to address these needs. VR enables the simulation of realistic social scenarios with high experimental control, reducing social anxiety by providing a safe, non-judgmental context for interaction (Didehbani et al., 2016). Previous research has consistently demonstrated that VR-based training can improve emotion recognition, social orientation, and conversational competence in both autistic children and adults (Lahiri et al., 2015; Lugrin et al., 2022). However, the effectiveness of these tools is often contingent on integrating robust pedagogical strategies and adaptive mechanisms that respond to users' internal states (Maddalon et al., 2024b).

A significant barrier to effective learning in autistic children is the difficulty in psychophysiological regulation (Hervas, 2017). Children on the autism spectrum often face challenges related to sustained attention, sensory processing, and arousal regulation—factors that can undermine the effectiveness of structured cognitive training programs (Case-Smith et al., 2015; Yun et al., 2014). When the cognitive demands of a task exceed the child's processing capacity, or when sensory input becomes overwhelming, it frequently leads to a dysregulation of physiological arousal (Jahromi et al., 2013; Pfeiffer et al., 2011). This dysregulation often manifests behaviorally as motor stereotypies, withdrawal, or frustration (Trevarthen and Delafield-Butt, 2013). To address this, adaptive support mechanisms during training have gained increasing attention for their ability to modulate user engagement and improve outcomes (Alcañiz et al., 2022; Maddalon et al., 2024b; Zahabi and Abdul Razak, 2020). Evidence suggests that well-timed pauses—whether rule-based, human-triggered (Wizard of Oz paradigm), or algorithm-driven—can reduce fatigue, sustain attention, and support emotion regulation across different populations (Biard et al., 2018; Kim et al., 2017; American Psychiatric Association DSMTF and American Psychiatric Association, 2013). In ASD-specific interventions, pauses are typically used for behavioral regulation (e.g., excessive verbalizations, stereotypies) rather than cognitive pacing and are often delivered by therapists rather than computers (Ahmad Lawan et al., 2023; LeBlanc et al., 2017). Research consistently demonstrates that structured pauses, including mindfulness, relaxation, and sensory tasks, outperform unstructured pauses that offer free time in both ASD and neurotypical groups (Cachia et al., 2016; Hatfield et al., 2023; Lang et al., 2011). Despite this promise, few studies integrating biosignals have compared different pause strategies. Most interventions rely solely on predefined behavioral triggers to administer pauses, limiting adaptivity and generalizability (Biard et al., 2018; Francese and Yang, 2022; Jyoti and Lahiri, 2020).

The present study embeds structured Adaptive Pauses (APs)—brief, strategically timed interruptions within the training protocol—designed to restore psychophysiological balance when activation levels (such as hyperactivation, hypoactivation, or emotional dysregulation) interfere with optimal engagement during the intervention. Unlike unstructured breaks, APs promote regulation, sustain engagement, and improve effectiveness through targeted activities such as guided breathing or sensory modulation (Billeci et al., 2016; Chiossi et al., 2025; Torrado et al., 2017). The theoretical rationale for implementing APs in this study is grounded in the Circumplex Model of Affect (Barrett and Russell, 1999; Russell and Barrett, 1999), which highlights the interplay between arousal and valence in shaping emotional experiences and behavioral responses. In ASD interventions, regulating arousal becomes particularly critical, as both heightened positive (i.e., overexcitement) and negative (i.e., frustration) states can equally disrupt task performance and learning (Case-Smith et al., 2015; Kushki et al., 2013). While hypoarousal often manifests as reduced engagement, attentional lapses or flattened affect, hyperarousal presents a unique diagnostic challenge: it may lead to withdrawal or even catatonic-like immobility (Case-Smith et al., 2015; Lane et al., 2012; Mazefsky et al., 2013). Because these behavioral cues are often subtle, counterintuitive, or present as immobility rather than overt agitation, traditional observation is frequently insufficient for accurate recognition (Mazefsky et al., 2013; Randell et al., 2022). To address these detection difficulties, interventions are increasingly integrating automated multimodal data collection—such as biosignals and behavioral tracking—which reduces human error and offers insights not observable through behavioral cues alone (Alcañiz Raya et al., 2020a; Maddalon et al., 2024b; Minissi et al., 2024; Yun et al., 2014). In the current design, APs utilize a guided breathing exercise combined with a sensory light component to foster relaxation and prevent cognitive and sensory overload. This approach, drawing on occupational therapy principles, promotes comfort through non-verbal, low-cognitive-demand stimulation, making it specifically accessible for autistic children who struggle with complex verbal instructions (American Occupational Therapy Association, 2008; Case-Smith and Arbesman, 2008; de Bruin et al., 2015; Jahromi et al., 2013). The use of adjustable lighting further supports behavioral regulation with minimal disruption to task flow (Fava and Strauss, 2010; Garzotto and Gelsomini, 2018; Mulas et al., 2019). While the AP content remained consistent, the administration method varied.

Building on a previously validated VR-based intervention for autistic children with level 1 (Maddalon et al., 2026), the present study extends this work by exploring differences across three AP implementation approaches—rule-based, Wizard of Oz, and automated-random model informed by biosignals—with a non-adaptive one. Participants were randomly assigned to one of four groups corresponding to these conditions. The adaptive system was pre-defined, and both behavioral and kinematic data were continuously collected, with APs introduced only at specific moments for usability reasons. Although biosignals were collected throughout the experimental sessions, the present study focuses on kinematic and behavioral data rather than physiological signals such as electrodermal activity. Physiological signal activity was collected as part of an exploratory component to support the development of the automated triggering algorithm. Previous work using the same setup demonstrated that electrodermal activity recordings were of insufficient quality for reliable analysis (Maddalon et al., 2026) due to data quality limitations, these signals were not included in this study. A detailed description of data processing and exploratory results is provided in the Supplementary material. Therefore, while acknowledging the theoretical importance of physiological monitoring for adaptive interventions, high-quality kinematic measures were prioritized to explore movement patterns, ensuring robust and interpretable results. This study had two primary objectives:

- To replicate and validate previous findings on the effectiveness of a VR social cognition program.
- To examine how different AP methodologies affect improvements in social-cognitive skills, as well as behavioral responses and kinematic patterns assessed during the intervention.

By integrating biosignals and AP design, this study aims to advance understanding of how personalized pacing in VR interventions can optimize learning outcomes and regulatory support in autistic children. Given the exploratory nature of the design, the study was not intended to provide definitive causal comparisons between adaptive strategies.

## 2 Materials and methods

### 2.1 Participants

Forty-six autistic children (aged 6–8 years) participated in this four-session study, including 38 males (*M* = 89.84 months, *SD* = 9.36) and 8 females (*M* = 94.87 months, *SD* = 7.58). The resulting male-to-female ratio of ~5:1 slightly exceeded the commonly reported 4:1 prevalence ratio (Loomes et al., 2017). Variation in group sizes was due to participant dropout (descriptive statistics in Table 1).

**Table 1:** Group characteristics and descriptive statistics summary.

Group	Triggering method	Maximum APs per session	N: males, females (M months, SD)
No-AP	None	0	12 males (*M* = 89.25, *SD* = 10.02)
Rule-based	Fixed after Games 2 and 4	2	10 males (*M* = 85.70, *SD* = 8.92), 1 female (*M* = 92.00)
Wizard of Oz	Human observer	6	5 males (*M* = 91.80, *SD* = 10.89), 5 females (*M* = 95.20, *SD* = 9.60)
Automated-random	Biosignal model (low accuracy)	6	11 males (*M* = 93.69, *SD* = 7.83), 2 females (*M* = 95.50, *SD* = 4.94)

Participants met DSM-5 criteria for ASD, Level 1 (requiring support), as determined by specialized psychiatrists integrating clinical records, Autism Diagnostic Observation Schedule, Second Edition (ADOS-2; module 2 or 3) findings, and adaptive behavior profiles (Vineland-II; Lord et al., 2012; American Psychiatric Association DSMTF and American Psychiatric Association, 2013). All participants had an IQ ≥ 85, measured with either the Wechsler Intelligence Scale for Children, Fifth Edition (WISC-V), or the Wechsler Preschool and Primary Scale of Intelligence, Fourth Edition (WPPSI-IV), depending on age (Wechsler, 2014, 2015). Complementary assessments of verbal comprehension, visuospatial skills, and memory were included to ensure suitability for the VR-based intervention. Three months prior to the intervention, the Child Behavior Checklist (CBCL/4–18; Achenbach, 1991) was used to screen for comorbidities; children with severe conduct disorders, severe anxiety, or epilepsy were excluded. Recruitment took place at Fundació Assistencial Mútua Terrassa (Ethical Committee; ID: P/23–111/), a specialized clinic for ASD in Spain. Caregivers provided informed consent, and participants received no monetary compensation.

### 2.2 Experimental groups

This study examined differences among adaptive methodologies integrated into a game-based intervention. Except for the no-AP group, all groups implemented APs with different triggering methods:

- **No-AP group**. Non-concurrent no-AP comparison group. This group was derived from a previously collected sample using the same intervention protocol (Maddalon et al., 2026). No APs were provided. It was not recruited concurrently with the other groups and therefore does not constitute a fully randomized control condition. As a result, comparisons involving this group should be interpreted as exploratory, as potential cohort or procedural differences cannot be fully controlled.
- **Rule-based adaptation group**. Based on prior observations (Maddalon et al., 2026) of the VR intervention's design, two key moments were identified when participants typically showed elevated activation (likely fatigue or frustration): after Games 1–2 and after Games 4–6. To avoid session overload, a maximum of two APs per session was scheduled—after Game 2 (motorically demanding) and Game 4 (requiring sustained attention and prone to inducing fatigue). This approach followed expert-designed, rule-based criteria built on expected response patterns.
- **Wizard of Oz adaptation group**. A trained experimenter observed activation states in real-time and determined AP timing, thereby simulating an automated system that ensures individualized delivery. A maximum of one AP per game (six per session) was allowed. Additional details regarding Wizard-of-Oz decision criteria for detecting elevated psychophysiological activation and model implementation are provided in the Supplementary material; key elements have been summarized in the main text to support reproducibility.
- **Automated-random adaptation group**. An exploratory machine learning model was initially developed to trigger automated APs from real-time biosignals. However, due to low accuracy (see Supplementary material), triggers were ultimately considered random. While the model triggered APs based on physiological activation, the rationale behind triggers remained unclear and did not reliably reflect participant state. Although the triggers were random, the group was retained to investigate whether random adaptation might still influence outcomes. This condition is therefore interpreted as a exploratory rather than a valid adaptive system. To limit fatigue and bias, no more than one AP per game was delivered.

### 2.3 VR-based intervention system and experimental procedure

Sociodemographic data and inclusion criteria were evaluated during the 3 months preceding the intervention. Pre-intervention assessments of social-cognitive skills were conducted approximately 2 weeks before the first training session.

The study employed a portable hybrid reality system embedded in a custom-designed semi-immersive environment (Maddalon et al., 2023a). The setup included an 86″ display, a computer running the VR software, and a desktop control panel for real-time monitoring. An Azure Kinect DK positioned beneath the display enabled full-body gesture tracking, projecting an unfilled virtual body that mirrored participants' real-time movements. Additionally, an EmotiBit wearable sensor provided non-invasive physiological monitoring (electrodermal activity and photoplethysmography), although these data were not analyzed in the present study. Sessions took place in a controlled environment (temperature: 24°C; stable lighting).

**Figure 1:**
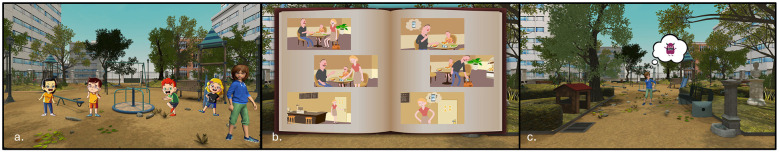
VR-based intervention. **(a)** Game 1. **(b)** Game 4. **(c)** Game 5.

The VR-based intervention, developed in Unity3D, consisted of six interactive games set in a virtual playground where participants engaged with a virtual human resembling a child (see Figure 1).

The intervention targeted specific sub-skills of social cognition aligned with the Theory of Mind framework:

- Game 1: emotion recognition
- Game 2: understanding that emotions are based on desires
- Game 3: spatial perspective-taking
- Game 4: first-order false belief
- Game 5: beliefs as triggers of emotions
- Game 6: second-order false belief

For details on the validation of game content, see previous publications (Maddalon et al., 2024a, 2026).

During each game, participants answered questions about the content by selecting responses through gesture-based interaction using their virtual bodies. In-game task difficulty increased progressively and was adjusted across sessions to match each participant's performance. If a participant failed to respond within 10 s, the virtual human repeated the question up to two additional times, creating a hint mechanism to sustain engagement and comprehension. Whenever an AP occurred, the experimenter ran the breathing activity, and the game was paused for the duration of the AP. Gameplay resumed exactly where it had been interrupted, 2 min later.

In the first session, participants completed a tutorial (Maddalon et al., 2023a, 2026) and chose a male or female virtual body to enhance self-recognition. Each session lasted approximately 1 h, with gameplay taking 20–25 min depending on the participant's pace and the occurrence of APs. The procedure was repeated across 1 weekly session to facilitate rest and consolidation of learning. Post-intervention assessments of social-cognitive skills were conducted approximately 2 weeks after the final session.

### 2.4 AP procedure

**Figure 2:**
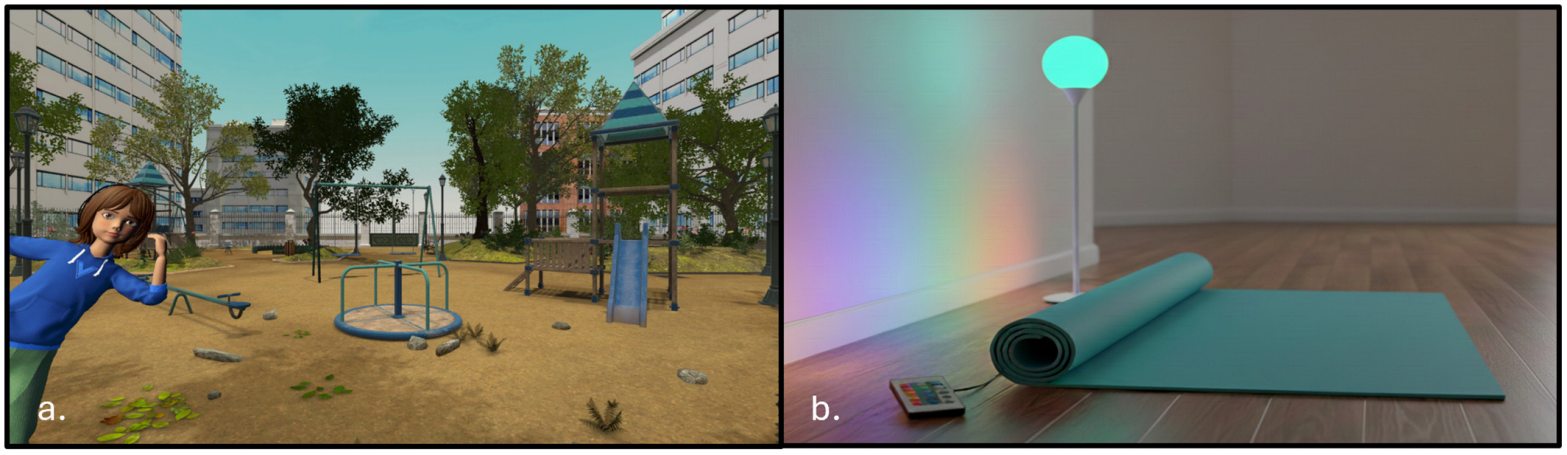
**(a)** Virtual human announces a break. **(b)** AP material representation (image generated via Gemini Pro).

When the AP triggered, the game was interrupted, and the child's virtual human announced a break (Figure 2a). Regardless of the adaptive method used, all APs followed the same experimental protocol and lasted approximately 2 min. The first minute was dedicated to introducing or briefly reminding participants of the activity, while the remaining time was spent on guided breathing relaxation. Breathing guidance was personalized to each child's ability and pace. The examiner monitored time via a hidden timer and ensured proper positioning before resuming gameplay.

During APs, the participant sat or lay on a provided mat while engaging in the breathing activity; simultaneously, a remote-controlled LED light was activated to provide sensory relaxation (Figure 2b). The examiner adjusted light colors and intensity in line with sensory regulation approaches, tailoring the choice to each participant's responses rather than following a fixed protocol. Although core elements of the AP protocol (duration, structure, and activity type) were standardized, some components—such as breathing pacing and light modulation—were individualized based on participant response. Formal intervention fidelity measures were not collected.

### 2.5 Measures

#### 2.5.1 Pre-post test measures

Social-cognitive skills were assessed pre- and post-intervention using two validated tools. The Theory of Mind Task Battery (ToMTB; Hutchins et al., 2008) is a standardized, performance-based test comprising 15 vignette-based questions of increasing complexity in Theory of Mind, directly aligned with the intervention's learning objectives. The Social Responsiveness Scale, Second Edition (SRS-2; Constantino et al., 2003), a 65-item caregiver-report questionnaire, assessed broader domains of social communication, social cognition, and repetitive behaviors associated with ASD. Together, these measures provided complementary perspectives on the intervention's effectiveness.

#### 2.5.2 Behavioral and kinematic measures

Behavioral data were collected via a custom Unity3D logging system that recorded participants' accuracy in each game and the number of hints used.

**Figure 3:**
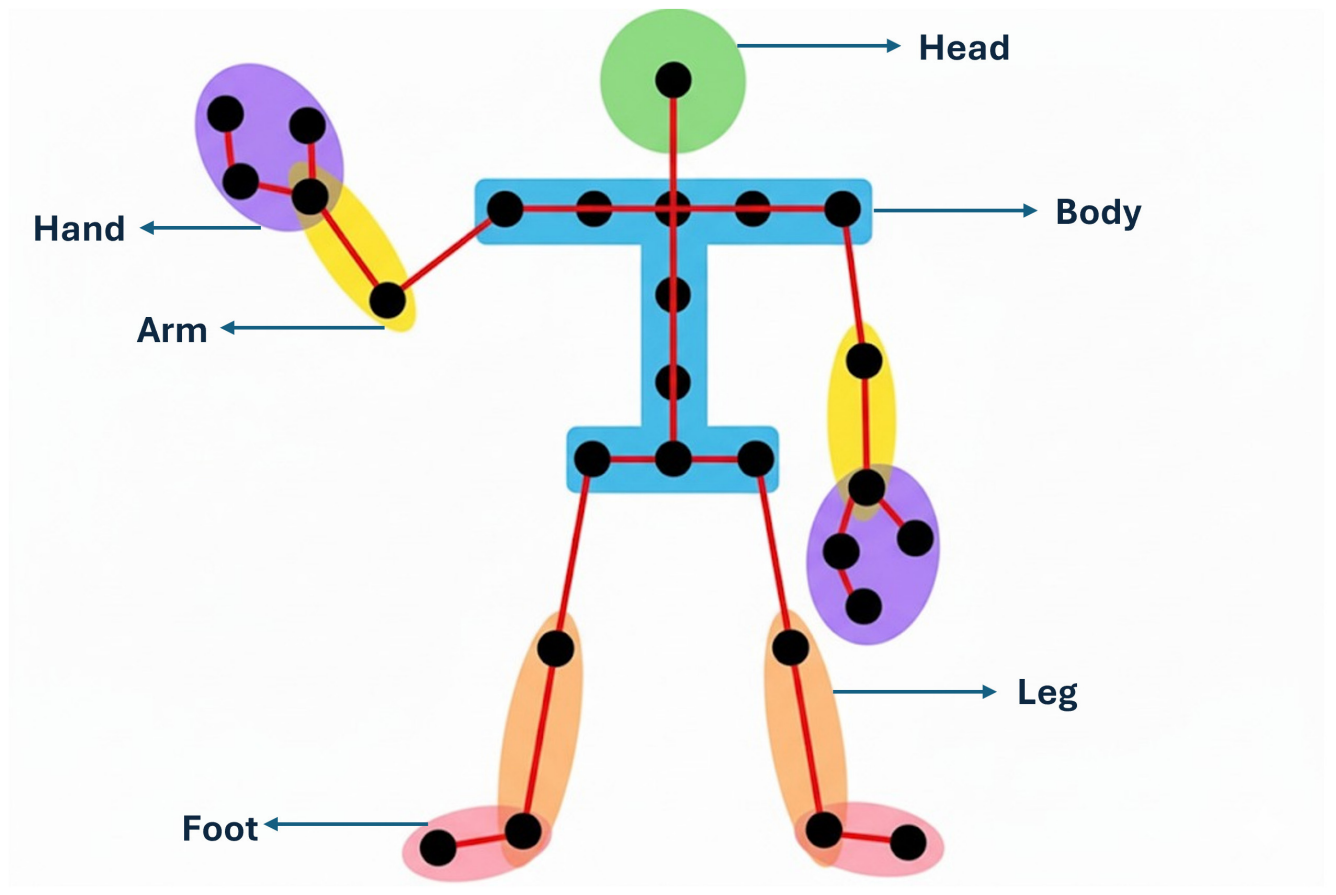
Body segmentation obtained through Azure Kinect DK sensor, showing detected joint positions (black dots) and corresponding anatomical body regions (color-coded).

Motor movement data were recorded for subsequent offline analysis of kinematic metrics. Positional data were obtained using the Azure Kinect DK, integrated with the Azure Kinect Body Tracking software and Unity3D. This setup enabled continuous tracking of 32 body joints. To ensure data quality and avoid redundancy due to high inter-variable correlation, 27 joints were selected for analysis, excluding facial markers (eyes, ears, and nose) and the neck. As illustrated in Figure 2, these joints were grouped into six anatomically meaningful body regions (head, body, arms, hands, legs, and feet), aggregating left and right sides into single regional metrics. This approach reduced dimensionality and offered a precise framework for analyzing motion patterns (see Figure 3).

### 2.6 Behavioral and kinematic data processing

Behavioral and kinematic data were processed to extract objective measures of participants' performance and motor behavior.

Behavioral data was processed to derive two primary metrics: response accuracy and number of hints used. Accuracy was calculated by coding individual responses as Correct (1) or Incorrect (0), while the number of hints captured how often the virtual human had to repeat the question for the participant to respond. Both metrics were computed per game across all six games.

Kinematic data underwent an initial visual inspection of the raw joint position data, revealing substantial noise likely due to sensor limitations. To address this, a moving-average filter (window size: five samples; Edwards and Green, 2014) was applied iteratively to produce smooth, differentiable position trajectories. Following data cleaning, two key metrics—mean acceleration and total displacement (both normalized by game duration)—were computed for each joint and grouped by body region (see Figure 3), as these measures have been shown to capture atypical motor patterns in ASD (Alcañiz Raya et al., 2020b; Cook et al., 2013; Minissi et al., 2024). Mean acceleration was obtained by applying two discrete derivatives to position data to derive 3D acceleration, computing the magnitude, and averaging over game duration by joint and body region. Total displacement was calculated as the sum of Euclidean distances between consecutive joint positions and averaged by body region.

### 2.7 Data analysis

A one-way ANOVA was conducted to determine whether there were any significant differences in age among the four experimental groups.

#### 2.7.1 Pre-post test analysis

Pre-post changes in social-cognitive measures were analyzed using a model-based approach. If the distribution of the dependent variables approximated a Gaussian or Gamma distribution, a Generalized Linear Mixed Model (GLMM) was employed. If these assumptions were not met, an aligned rank transform (ART) procedure was used. The model formula was:


$$\begin{array}{l} \text{Outcome }\sim \text{ PrePost}*\text{ Group }+\;(1|\text{Participant}) \end{array}$$

For ToMTB, a Gamma distribution with a log link was used. For SRS-2, a Gaussian distribution with identity link was used. Where significant effects were detected, Estimated Marginal Means (EMM) were computed to assess the significance of pairwise contrasts. To investigate the reproducibility and validity of previous findings, significant pre-post differences in social-cognitive measures were tested using overall pre-post contrasts, marginally averaged over Group. To explore the impact of the different AP methodologies, it was analyzed whether pre-post differences varied across the four groups using group-wise pre-post contrasts.

#### 2.7.2 Behavioral and kinematic analyses

A similar framework, aligned with the objective, has been used to analyze behavioral and kinematic outcomes across sessions and groups. After validating model assumptions (with a Type I error threshold of 0.01), appropriate distributions and link functions were used: a Gamma with a log link for positive, continuous metrics (e.g., kinematic features: mean acceleration and total displacement), Poisson with log link for count-based metrics (e.g., number of hints used), or binomial with log link for binary outcomes (e.g., response accuracy).

Outliers in motor movement data were removed prior to analysis using the interquartile range (IQR) method (λ = 1.5 × IQR) to reduce the influence of extreme values. Twelve GLMMs were fitted to assess changes in movement across sessions for mean acceleration and displacement of the head, body, arms, hands, legs, and feet.

The following model structure was applied to all behavioral and kinematic variables:


$$\begin{array}{l} \text{Outcome }\sim \text{ Session}*\text{ Group }+\;(1|\text{Participant})\;+\;\left(1|\text{Game}\right) \end{array}$$

Models included fixed effects for session, group, and their interaction, with random intercepts for participant and game to account for individual variability.

#### 2.7.3 Multiple comparison correction

To mitigate the risk of Type I error inflation, the Holm-Bonferroni correction was applied to the kinematic outcomes as a single family of tests. This adjustment was applied because multiple correlated metrics extracted from motor movement data address a shared hypothesis regarding physical activity. Notably, this multiple-comparison correction was not applied to behavioral metrics or to the pre-post questionnaire analyses, as these represent distinct outcome domains (e.g., social-cognitive skills, or task-specific accuracy) and were treated as separate research questions, each involving a limited number of planned comparisons. This approach is consistent with standard statistical practice when outcomes are theoretically independent and not part of the same inferential family. These tests were therefore addressed as independent research questions to differentiate between the effects of session, group, and their interaction.

Additionally, when significant effects were detected, *post-hoc* comparisons were conducted to identify specific differences. EMMs were computed, and pairwise comparisons were performed, with the resulting *p*-values adjusted using the Tukey method to account for multiple comparisons within the family of estimates.

## 3 Results

Participants in the four groups did not differ in age, $\chi_{\left(3\right)}^{2}$ (*N* = 46) = 5.42, *p* = 0.14.

Exploratory inspection of pause delivery indicated that the number of pauses per participant was generally low across sessions. In the Wizard-of-Oz condition, pauses were infrequent and often limited to one occurrence in selected sessions, while in the automated condition most participants received approximately one pause per session.

### 3.1 Pre-post test results

Analysis of the ToMTB scores revealed a significant main effect of Pre-post [χ^2^_(1)_ = 4.06, *p* = 0.043], indicating overall improvement across the sample. Specifically, the main effect contrasts showed that Post-test scores were significantly higher (*EMM* = 2.42, *SE* = 0.034) than Pre-Test scores (*EMM* = 2.32, *SE* = 0.034), confirming an overall improvement (*z-ratio* = 2.979, *p* = 0.0029). Additionally, a significant main effect of Group was observed [χ^2^_(3)_ = 11.06, *p* = 0.011], but the change over time did not differ across groups, as indicated by a non-significant Pre-Post × Group interaction [χ^2^_(3)_ = 0.66, *p* = 0.88].

In contrast, the SRS measure showed no significant effects. Neither the main effect of Pre-Post [χ^2^_(1)_ = 1.79, *p* = 0.180], the main effect of Group [χ^2^_(3)_ = 0.83, *p* = 0.842], nor for the Pre-Post × Group interaction [χ^2^_(3)_ = 2.60, *p* = 0.457] reached statistical significance.

### 3.2 Behavioral results

Analysis of accuracy showed a marginal main effect of Session [χ^2^_(3)_ = 7.59, *p* = 0.055], suggesting a potential trend toward improvement over time, although the effect did not reach statistical significance. *Post-hoc* comparisons showed that accuracy was significantly higher in Sessions 3 (S3) and 4 (S4) than in Session 1 (S1). Furthermore, S3 and S4 exhibited significantly greater accuracy than session 2 (S2), confirming a robust learning curve. In contrast, neither a main effect of Group [χ^2^_(3)_ = 0.93, *p* = 0.816], nor a Session × Group interaction [χ^2^_(9)_ = 5.22, *p* = 0.813] was found.

**Figure 4:**
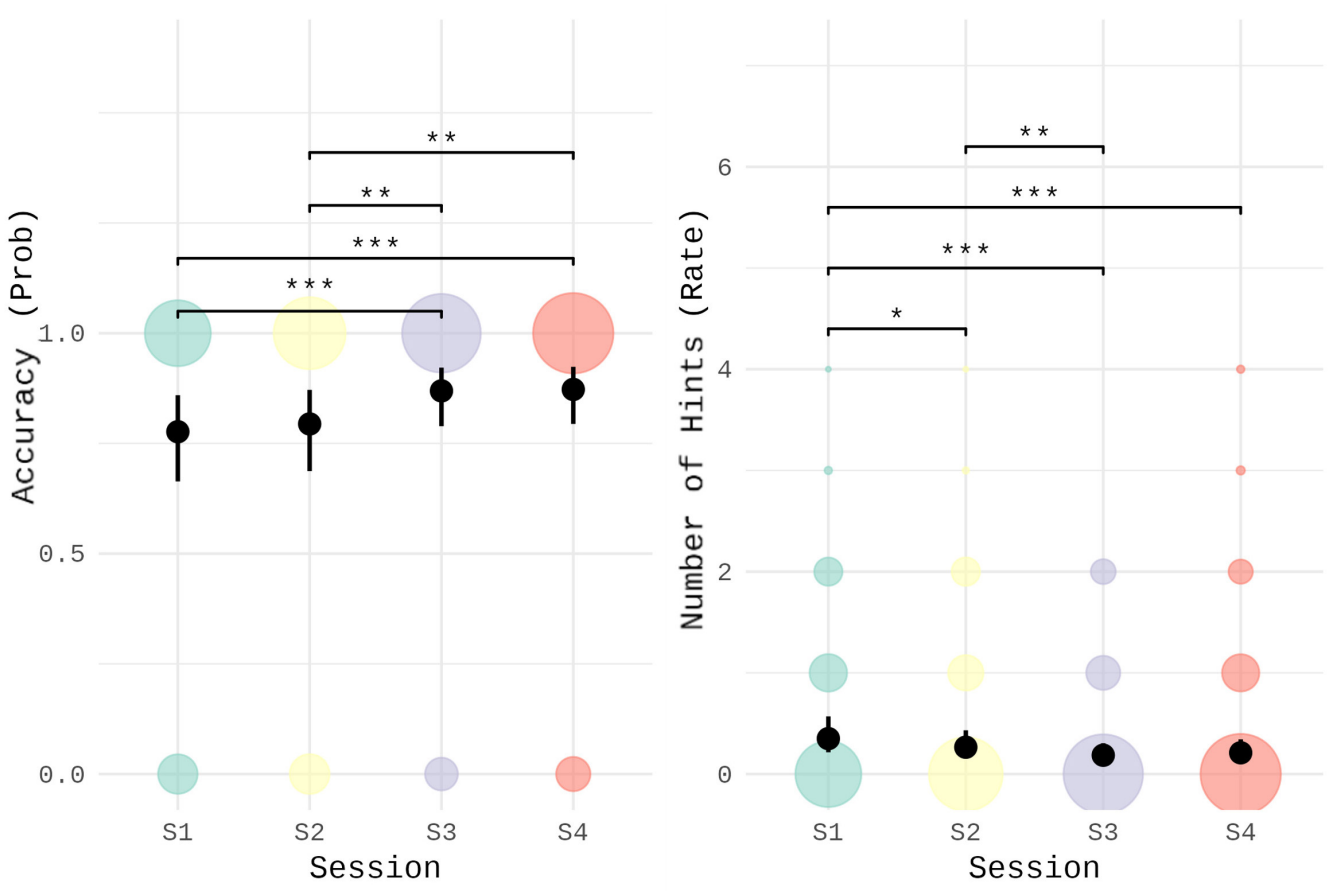
Distribution of behavioral metrics by session (accuracy distribution: 1 = correct, 0 = incorrect). Violin plot widths represent data density, while internal markers indicate session means and variability. Statistically significant pairwise differences are indicated by symbols: (*) for *p* < 0.05, (**) for *p* < 0.01, and (***) for *p* < 0.001.

The analysis of the number of hints revealed a significant main effect of Session [χ^2^_(3)_ = 9.26, *p* = 0.025]. *Post-hoc* comparisons indicated a general reduction in the number of hints required across the sessions. Specifically, participants required significantly more hints in S1 than in S2, S3, and S4. Furthermore, the number of hints was significantly higher in S2 compared to S3. No significant main effect of Group [χ^2^_(3)_ = 3.15, *p* = 0.368] or Session × Group interaction [χ^2^_(9)_ = 6.88, *p* = 0.649] was observed. Adjusted *p*-values for the contrasts are visualized in Figure 4 (refer to Supplementary material for the full suite of significant results).

### 3.3 Kinematic results

**Figure 5:**
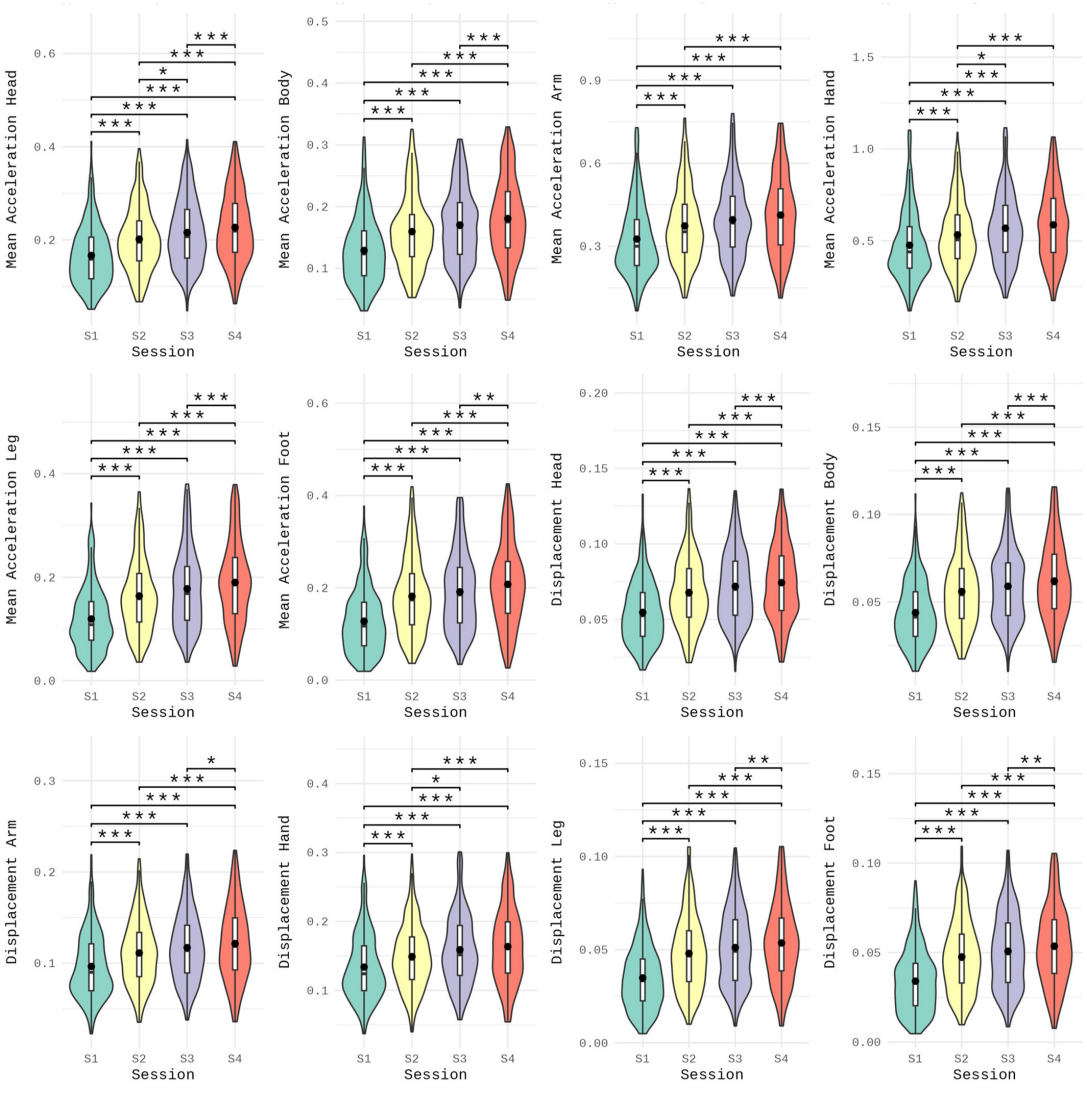
Distribution of kinematic metrics by session. Violin plot widths represent data density, while internal markers indicate session means and variability. Statistically significant pairwise differences are indicated by symbols: (*) for *p* < 0.05, (**) for *p* < 0.01, and (***) for *p* < 0.001.

Results are reported at the body-region level, with left and right joints collapsed into a single metric per region. Analysis of the 12 Kinematic measures confirmed a highly significant main effect of Session for all metrics, even after correction. However, no main Group or Session × Group interaction effects survived the correction for multiple comparisons. *Post-hoc* contrasts revealed a consistent pattern across sessions, with S1 displaying significantly lower acceleration and displacement than S2, S3, and S4. During training, S2 showed lower acceleration and displacement metrics than S4. Furthermore, S3 generally displayed significantly lower acceleration and displacement metrics than S4, and in a few specific instances, lower values than S2. Comprehensive statistics for main and interaction effects are provided in Table 2, while adjusted *p*-values for the contrasts are visualized in Figure 5 (refer to Supplementary material for the full suite of significant results).

**Table 2:** Omnibus statistical results for kinematic metrics across sessions and groups.

Outcome	Effect tested	Statistic	Adjusted *p* (Holm-Bonferroni)
Kinematic mean acceleration measures			
Head	Session main effect	χ^2^_(3)_ = 87.11	< 0.0001^***^
	Group main effect	χ^2^_(3)_ = 3.865	NA(†)
	Interaction effect	χ^2^_(9)_ = 9.69	NA
Body	Session main effect	χ^2^_(3)_ = 92.34	< 0.0001^***^
	Group main effect	χ^2^_(3)_ = 8.66	>0.05
	Interaction effect	χ^2^_(9)_ = 15.52	NA
Arm	Session main effect	χ^2^_(3)_ = 56.18	< 0.0001^***^
	Group main effect	χ^2^_(3)_ = 3.21	>0.05
	Interaction effect	χ^2^_(9)_ = 13.53	NA
Hand	Session main effect	χ^2^_(3)_ = 47.52	< 0.0001^***^
	Group main effect	χ^2^_(3)_ = 2.98	NA
	Interaction effect	χ^2^_(9)_ = 14.34	NA
Leg	Session main effect	χ^2^_(3)_ = 102.2	< 0.0001^***^
	Group main effect	χ^2^_(3)_ = 9.21	>0.05
	Interaction	χ^2^_(9)_ = 23.26	>0.05
Foot	Session main effect	χ^2^_(3)_ = 99.96	< 0.0001^***^
	Group main effect	χ^2^_(3)_ = 9.37	>0.05
	Interaction	χ^2^_(9)_ = 24.632	>0.05
Kinematic displacement measures			
Head	Session main effect	χ^2^_(3)_ = 85.29	< 0.0001^***^
	Group main effect	χ^2^_(3)_ = 4.927	NA
	Interaction effect	χ^2^_(9)_ = 5.74	NA
Body	Session main effect	χ^2^_(3)_ = 95.55	< 0.0001^***^
	Group main effect	χ^2^_(3)_ = 8.071	>0.05
	Interaction effect	χ^2^_(9)_ = 8.209	NA
Arm	Session main effect	χ^2^_(3)_ = 59.35	< 0.0001^***^
	Group main effect	χ^2^_(3)_ = 2.72	NA
	Interaction effect	χ^2^_(9)_ = 10.26	NA
Hand	Session main effect	χ^2^_(3)_ = 45.79	< 0.0001^***^
	Group main effect	χ^2^_(3)_ = 1.88	NA
	Interaction effect	χ^2^_(9)_ = 12.02	NA
Leg	Session main effect	χ^2^_(3)_ = 109.99	< 0.0001^***^
	Group main effect	χ^2^_(3)_ = 9.07	>0.05
	Interaction effect	χ^2^_(9)_ = 14.26	NA
Foot	Session main effect	χ^2^ = 110.24	< 0.0001^***^
	Group main effect	χ^2^ = 8.99	>0.05
	Interaction	χ^2^ = 16.745	NA

(†)NA, not applicable. Statistically significant results are indicated by symbols: ^*^p < 0.05, ^**^p < 0.01, ^***^p < 0.001.

## 4 Discussion

### 4.1 Summary of main findings

This study builds on prior work on VR-based adaptive training for autistic children, focusing on the role of AP methodologies in shaping social-cognitive, behavioral, and kinematic outcomes.

Pre-post analyses confirmed significant improvements in Theory of Mind as measured by ToMTB, while SRS-2 scores remained unchanged—a finding consistent with its broader caregiver-report format, which captures generalized social functioning rather than direct assessment of targeted cognitive skills. The absence of significant changes in SRS-2 scores suggests that improvements may be limited to trained social-cognitive processes and may not readily translate to broader, caregiver-reported social functioning. The observed improvement in ToMTB performance is consistent with previous findings using the same VR intervention, providing convergent evidence for its effectiveness across independent samples. As in the previous study, session progression emerged as the strongest predictor of improvement across the study's core metrics, highlighting the combined effects of task familiarization and motor learning. Accuracy trended upward across sessions, and reliance on hints decreased progressively, suggesting enhanced task efficiency and growing independence in social-cognitive processing. Moreover, kinematic analyses revealed significant session effects on movement efficiency; specifically, acceleration and displacement metrics showed a progressive stabilization, suggesting more purposeful and regulated motor control during social-cognitive tasks. Due to low statistical power it is advisable to consider that observed session-related improvements may reflect multiple mechanisms, including practice effects and task familiarization, rather than specific effects of the intervention or AP strategies.

Contrary to expectations, AP methodologies did not yield distinct outcome patterns across experimental conditions. Taken together, these results suggest that the core VR-based intervention itself, rather than the specific AP strategy employed, accounted for the observed therapeutic gains. Importantly, the study does not provide evidence for differential effects of AP, although alludes that structured APs are feasible, safe, and yield results no different from those of the non-concurrent comparison condition, providing an empirical basis for further methodological optimization and theoretical development of real-time adaptive systems.

### 4.2 Feasibility and implications for adaptive systems

#### 4.2.1 Social-cognitive outcomes and learning trajectories

The study confirmed earlier findings of significant improvements in ToMTB post-test scores, indicating that VR-based training enhanced social-cognitive skills in autistic children. These gains, observed after just four sessions, likely stem from the adaptive nature of VR environments, which allow children to practice interpreting social cues in controlled contexts calibrated to individual progress levels (Bravou et al., 2022; Frolli et al., 2022; Maddalon et al., 2023b). This adaptive scaffolding may reduce extraneous cognitive load while promoting gradual skill consolidation through distributed practice (Maddalon et al., 2024b; Calderone et al., 2024). The brief intervention period suggests that even time-limited VR exposure can yield measurable improvements when training is systematically structured and individually responsive.

Although neither the main effect of group nor the interaction effect was statistically significant, the absence of detrimental effects across all AP conditions indicates that structured pauses can be integrated into VR interventions without undermining therapeutic progress. This finding is clinically relevant, as it establishes the safety profile of AP methodologies while leaving open the question of optimal implementation. Future adaptive systems should therefore continue to investigate which specific AP parameters (e.g., duration, trigger thresholds, and activity type during pauses) maximize engagement and learning efficiency.

While most outcome metrics confirmed earlier findings, some discrepancies warrant further interpretation. Task accuracy narrowly missed statistical significance across sessions (*p* = 0.055), likely due to heterogeneity in individual response patterns and the limited sample size, which reduced statistical power to detect modest, clinically meaningful effects. Although not reaching significance thresholds, examination of estimated marginal means and pairwise contrasts revealed a pattern consistent with the previous study. Results indicated an upward trajectory in accuracy as the sessions progressed, with S1 and S2 exhibiting lower performance than the later stages of training (S3 and S4), during which children demonstrated advances in socio-cognitive task completion. This delayed emergence of gains may reflect the gradual internalization of practiced skills and increased response confidence, both likely fostered by the safe, non-judgmental nature of the VR environment (Maddalon et al., 2026). Such environments are known to reduce social performance anxiety and encourage exploration of novel interaction strategies without fear of real-world social consequences (American Psychiatric Association DSMTF and American Psychiatric Association, 2013; Didehbani et al., 2016; Kourtesis et al., 2023).

The progressive reduction in reliance on hints further supports this interpretation. As task complexity increased across sessions, participants paradoxically required less external support, which might indicate genuine skill mastery rather than simple task repetition effects. This pattern aligns with Vygotsky's zone of proximal development and with Wood et al.'s (1976) scaffolding framework, in which instructional support is systematically withdrawn as learner competence grows. The observed reduction in scaffolding needs suggests that participants developed both procedural mastery and metacognitive confidence—critical components of skill generalization beyond the training context (Al-Hamdani and Yousif, 2025).

Contrary to expectations, no significant differences between-group differences emerged in session-level performance metrics, likely due to insufficient statistical power given the modest sample size. However, the within-subjects learning trajectories were robust and consistent, suggesting that the core intervention mechanisms operate reliably across individuals in enhancing social-cognitive skill acquisition through repeated, structured exposure, regardless of AP condition.

#### 4.2.2 Kinematic markers of motor learning and behavioral adaptation

Concordant with the previous study, kinematic analyses revealed an ascending trend in both acceleration and spatial displacement across all measured body segments. Specifically, motor activity increased progressively from S1 through S4, indicating robust session-driven increases. These changes likely reflect task familiarization or reduced uncertainty, motor learning consolidation, and reduced movement inhibition. Over successive sessions, participants exhibited sharper accelerations, greater spatial excursions, and more fluid movements—a pattern consistent with prior findings on kinematic signatures suggesting increased confidence, environmental comfort, and self-efficacy within the VR intervention space (Hocking et al., 2022; Jyoti and Lahiri, 2022; Zhao et al., 2018).

These motor changes align theoretically with exposure therapy principles, wherein repeated, structured encounters with anxiety-provoking stimuli reduce avoidance behaviors and hyperarousal (Maskey et al., 2014). For autistic children, who frequently display motor rigidity, restricted movement patterns, and heightened behavioral inflexibility in novel environments (Wallbridge et al., 2024), the observed kinematic progression represents meaningful behavioral adaptation. The gradual increase in movement amplitude and velocity suggests that initial motor constraint—potentially reflecting anxiety, uncertainty, or sensory overwhelm—diminished as children acclimated to the virtual environment and task demands (Maddalon et al., 2026).

Notably, these kinematic improvements occurred independently of AP condition, further supporting the conclusion that core intervention effects (repeated exposure, task structure, and adaptive difficulty) drive primary outcomes. However, the sensitivity of kinematic measures to detect within-session changes positions them as potentially valuable real-time indicators for future adaptive systems. Movement patterns could serve as objective biomarkers of engagement, arousal, or cognitive load, complementing or potentially replacing subjective behavioral observations.

#### 4.2.3 Toward intelligent adaptive systems: biosignal integration and machine learning

Looking forward, biosignal-driven adaptations represent a promising yet underexplored frontier in personalized VR interventions. Physiological metrics (e.g., heart rate variability, electrodermal activity, and respiratory rate) and kinematic data can capture moment-to-moment fluctuations in arousal, attention, and affective state that may be difficult or impossible to discern through behavioral observation alone (Alcañiz Raya et al., 2020a; Minissi et al., 2024). Such continuous, objective measurement could enable more precise, individualized intervention adjustments that respond dynamically to children's regulatory needs (Maddalon et al., 2024b).

To more accurately evaluate AP methodologies, future studies should implement fully automated AP systems powered by multimodal biosignals and machine learning algorithms. The exploratory automated-random model tested in the current study (implemented for one experimental group) lacked the predictive accuracy required to reliably detect arousal states necessitating intervention. This outcome is primarily attributable to three critical challenges:

- *Data Integrity*: Inherent signal noise and motion artifacts are pervasive when recording from active pediatric populations with ASD/ADHD (Kowallik and Schweinberger, 2019). This is particularly evident in Galvanic Skin Response data; while clinical standards assume a resting state, the high degree of motor engagement in our VR protocol led to significant signal degradation.
- *Methodological Complexity*: Developing real-time predictive models from heterogeneous, time-series biosignals is fraught with difficulty. This is exacerbated by highly imbalanced class distributions—where high-arousal events are statistically rare—and by the reliance on expert-annotated ground truth labels, which are inherently subjective.
- *Construct Multi-dimensionality*: High activations (e.g., frustration, agitation, or over-excitement) are multifaceted and difficult to classify as a single discrete label.

Rather than attempting to predict high-level states from scratch, a more robust strategy involves a bottom-up multimodal approach (Friston et al., 2014). This entails first detecting lower-level markers—such as stereotypical motor patterns or sharp electrodermal spikes—before inferring complex psychological constructs. For example, increased arousal is not exclusively linked to a single construct; modeling these foundational behaviors is essential, despite the significant research challenges each poses.

Despite these limitations, the exploratory model tested here represents a vital step toward personalized, real-time adaptive systems (see Supplementary material for further details on model implementation). Such systems hold immense potential for the autistic population, who often exhibit atypical arousal patterns, impaired self-regulation, and difficulties in communicating internal states (Case-Smith et al., 2015; Kushki et al., 2013). Future iterations should prioritize integrating robust biosignal processing pipelines with machine learning architectures capable of high-fidelity, real-time state classification. Specifically, systems should incorporate:

1. Multimodal sensor fusion: combining physiological, kinematic, and behavioral (gaze, facial expression, task performance) data streams to create comprehensive arousal and engagement profiles;
2. Individualized baseline modeling: establishing person-specific normative ranges and response patterns during initial calibration sessions to account for high inter-individual variability in ASD populations;
3. Context-aware algorithms: developing models that distinguish between adaptive arousal (task engagement) and maladaptive arousal (distress, overload) by incorporating task context, performance trends, and temporal dynamics.

Such architectures could ultimately enable autonomous VR-agents capable of tailoring interaction pace, complexity, and support level to each child's real-time regulatory state (Zhao et al., 2026). This level of personalization could substantially enhance both intervention feasibility (by reducing clinician burden) and clinical impact (by optimizing the therapeutic dose and timing of each interaction component).

#### 4.2.4 Summary of implications

Overall, findings highlight both the therapeutic promise and current technical limitations of AP-enhanced VR interventions. The core VR platform demonstrably facilitates socio-cognitive skill acquisition and motor behavioral adaptation, while structured APs—at the very least—do not impede progress and establish feasibility for integration. The central challenge now lies in advancing from feasibility to optimality: developing intelligent pause mechanisms that not only respond to dysregulation but also actively optimize engagement trajectories, prevent cognitive overload before it occurs, and maximize the generalization of learned skills to naturalistic social contexts.

Pre- and post-intervention clinical questionnaires, along with behavioral and kinematic data, indicate that the intervention successfully supported progressive increases in social task complexity, enabling participants to tackle more challenging tasks. These findings suggest that immersive VR, when combined with structured therapeutic principles, can effectively advance clinical goals for autistic children by fostering learning in low-stakes, controlled contexts while simultaneously reducing characteristic motor rigidity and inflexibility.

### 4.3 Limitations and future directions

Several methodological and conceptual limitations must be acknowledged to properly contextualize findings and guide subsequent research.

First, the relatively small sample size reduced statistical power to detect subtle group × session interactions, thereby limiting the ability to draw definitive conclusions about the comparative efficacy of different AP methodologies. As a result, the absence of significant group differences should not be interpreted as evidence of equivalence between AP conditions but rather as inconclusive. While the main effects of the session were robust for the social-cognitive (ToMTB), behavioral, and kinematic measures, between-group comparisons may have been underpowered to detect modest but clinically meaningful differences in AP approaches. While no differences between AP conditions were observed, these comparisons should be interpreted cautiously due to design limitations, including a non-concurrent comparison group, variation in pause frequency across conditions, and limited statistical power. The findings primarily support the feasibility of integrating structured pauses into VR-based interventions. Moreover, differences in sex distribution across groups may have introduced additional variability. As a result, non-significant group differences should not be interpreted as evidence of equivalence between adaptive pause conditions.

Second, APs were triggered primarily in response to medium-to-high arousal states, potentially neglecting attentional disengagement or more subtle regulatory needs. This threshold-based approach may have missed opportunities for preemptive support or failed to address the full spectrum of dysregulation patterns common in autistic populations. This potential misalignment between observable behavior and internal state underscores the necessity of objective biosignal measurement for accurate state detection.

Third, differences in pause frequency across conditions, the use of non-concurrent comparison group, and the exploratory nature of the automated-random condition, limited the interpretability of the group comparisons. This condition primarily served as a proof-of-concept rather than a fully validated intervention approach, and the results should be interpreted within this developmental context. Importantly, the study design does not allow full separation of adaptation strategy from pause frequency. However, exploratory inspection of pause delivery suggests that the effective number of pauses per session was generally low and less variable across groups than the theoretical maximum would suggest. Nevertheless, this confound cannot be ruled out and limits causal interpretation.

Finally, evidence for generalization beyond the training context is limited. While improvements were observed in task-specific social-cognitive measures (ToMTB), no significant changes were detected in caregiver-reported outcomes (SRS-2). Additionally, no follow-up assessment was conducted, preventing evaluation of whether observed gains persist over time or transfer beyond the laboratory setting.

Based on these limitations, some directions for future investigation emerge. First, larger sample sizes are needed to achieve adequate power to detect group × session interactions and to confirm the preliminary trends observed here. Second, future work should develop and validate real-time adaptive systems that adjust intervention pacing based on multimodal biosignal thresholds, incorporating cardiovascular, electrodermal, kinematic, and behavioral data streams with validated state classification algorithms. Finally, examining individual differences that may moderate AP effectiveness, including sensory sensitivities, to identify subgroups who benefit most from specific adaptive approaches.

### 4.4 Conclusion

This study confirmed the effectiveness of brief VR-based social cognition training for autistic children and demonstrated the robustness of behavioral and kinematic outcome measures across repeated sessions. While different AP methodologies produced comparable therapeutic outcomes, the findings establish the feasibility and safety of integrating structured pauses into immersive interventions. Importantly, the absence of negative effects across all AP conditions provides a foundation for continued methodological development without concern for harm. However, conclusions regarding the comparative efficacy of AP strategies remain preliminary due to design and statistical limitations.

Adaptive systems powered by multimodal biosignals and machine learning remain a promising direction for next-generation personalized interventions. With continued refinement—particularly in sensor integration, algorithm development, and individualized modeling—intelligent virtual agents could deliver optimally timed, individually calibrated pacing adjustments that maximize engagement, minimize overload, and promote sustainable learning in ASD populations. The path forward lies in transitioning from feasibility demonstration to precision optimization, leveraging computational advances to fully realize the therapeutic potential of adaptive immersive technologies.

## Data Availability

The raw data supporting the conclusions of this article will be made available by the authors, without undue reservation.
