# Supplementary material for: Behavioral and kinematic outcomes of adaptive pauses in VR social cognition training for autistic children
Source: Front Psychol. 2026 May 29;17:1827283. doi: 10.3389/fpsyg.2026.1827283 (PMC13260389; doi:10.3389/fpsyg.2026.1827283)
Supplement: Supplementary file 1 [file Data_Sheet_1.docx]

Supplementary Material

# Wizard of Oz adaptation group and criteria used to detect activation episodes

Relying on the theoretical framework of the Circumplex Model of Affect (Barrett & Russell, 1999; Russell & Barrett, 1999), the experimenter—trained to recognize states of psychophysiological activation with positive or negative affect—determined the occurrence and timing of APs during the session. The specific criteria for identifying episodes of heightened activation are outlined in Supplementary Table 1.

**Supplementary Table 1**. Guidelines for AP Intervention.

| **Negative Valence Activation** | - Motor stereotypies involving hands, arms, or the entire body. Physical hyperactivation (e.g., repetitive movements, increased frequency of stereotypies). - Shift to hypoactivation (e.g., lying on the floor, freezing in place). Verbally silent—minimal vocalizations or verbalizations. Lack of facial expression (blank face), absence of body gestures. Non-responsive behavior—appears not to listen, avoid eye contact, fail to initiate interactions, disregard instructions, or ignore adults. - Play refusal (e.g., verbal or physical resistance—throwing oneself to the ground, avoiding looking at the screen). - Muscle tension - Increased sensory sensitivity: persistent discomfort with devices (e.g., touching/fidgeting with a bracelet or glasses), irritation from sensory stimuli. - Verbal complaints expressing anger or discomfort - Verbal stereotypies: screaming or vocalizations. |
| --- | --- |
| **Positive Valence Activation** | - Physical hyperactivation to express happiness (e.g., running in place, jumping, hand-flapping, rocking movements). Motor stereotypies with bodily hyperactivity, sometimes accompanied by joyful sounds or verbalizations. - Non-verbal expressions of joy: excessive smiling, hugging, or affectionate gestures. |

# Automated-random adaptation group and exploratory machine learning

Model for Real-Time Adaptive Pause Delivery Based on Biosignals.

This section describes the development and implementation of an exploratory machine learning (ML) model designed to deliver Adaptive Pauses (APs) in real time, based on the analysis of physiological and kinematic signals. The objective of the model is to detect moments of heightened psychophysiological activation in children during VR training and to trigger an AP aimed at reducing stress or overstimulation. The model was developed through the following stages:

1. Labeling of training data;
2. Signal acquisition and input data preparation;
3. Dataset construction;
4. Model training;
5. Real-time model inference;
6. Model validation;
7. Integration into the VR training environment.

## Labeling

To train the model, it was necessary to collect data segments corresponding to moments when a child required a pause due to negative or intense activation. These moments were identified through a video labeling procedure based on a prior study (N = 23; Maddalon et al., 2026 submitted manuscript), which was conducted under identical experimental conditions. Three trained psychologists independently reviewed session videos and annotated moments of increased psychophysiological arousal using a shared definition informed by the Circumplex Model of Affect (Russell & Barrett, 1999; Barrett & Russell, 1999; Supplementary Figure 1). The labeling focused on both valence (positive, negative, neutral) and arousal intensity.


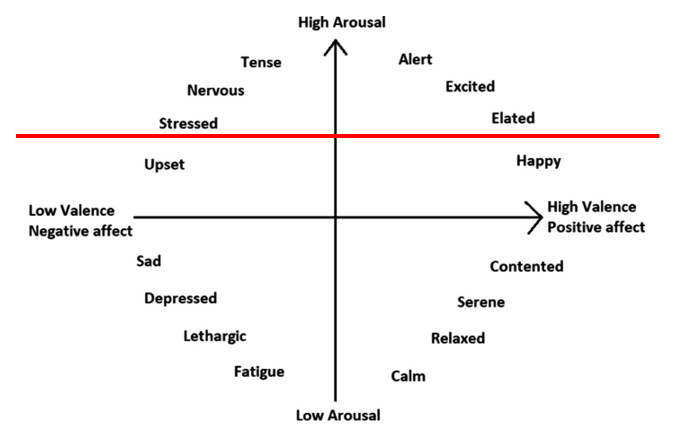


**Supplementary Figure 1**. Circumplex Model of Affect in valence and arousal dimensions.

The labeling task involved analyzing the child's behavior at an explicit level (e.g., frustration, motor agitation, or excitement) to detect moments of moderate-to-high arousal activation (red cutoff line in Figure 1) and align with implicit signals. Only events lasting approximately 10 seconds were selected to ensure sufficient signal duration for real-time analysis.

A pre-labeling calibration session was held to align the psychologists on the criteria for identifying medium-to-high psychophysiological activation. They jointly analyzed 10 videos and agreed on a shared annotation strategy. Example annotations include:

- “Child is motorically agitated, singing and dancing while playing with their hair, ignoring the task.”
- “Child reacts to success with repeated leg-kicking, scratching, strange facial expressions, and clapping.”

To ensure standardization, the following protocol was adopted:

1. Activation episodes were logged in structured tables per user and session, including start/end times and valence.

2. Each episode was analyzed based on four criteria:

- There is a clear activation.
- The activation persists for at least 7 seconds.
- The activation intensity is sufficient to justify a pause.
- Specify the valence (positive, negative, neutral).

An agreement between at least two of the three psychologists was required for an episode to be considered valid. Out of 100 reviewed videos, approximately 60 activation events were identified, of which 56 reached agreement and were used to define training targets for the ML model.

## Input Data

Three biosignal types were recorded for model training:

- Galvanic Skin Response (GSR): Acquired via EmotiBit at 10 Hz.
- Heart Rate (HR): Includes Photoplethysmography in Infrared (PI), Red (PR), and Green (PG) channels, also recorded via EmotiBit at 10 Hz.
- Kinematic Data: Full-body joint data (32 joints, 20 selected), recorded via Microsoft Kinect at 15 Hz in 3D space (X, Y, Z).

All signals were timestamped and temporally synchronized with the labeled events. Labels were binarized into two classes: activated and not activated. An instance was considered activated if at least two psychologists had independently labeled it as such, regardless of valence.

## Dataset Construction

A sliding-window approach was employed to segment each raw signal into 15-second windows. If a window overlapped with an activated label, it was tagged accordingly. Both positive and negative high-arousal episodes were included under the activated label (Supplementary Figure 2).

- Final dataset: ~11,800 not activated windows and ~250 activated windows.
- Raw GSR values >100µS were excluded as potential artifacts; zero values were retained.
- HR windows included PI, PG, and PR values.
- Kinematic windows included 60 values (20 joints × 3 spatial body dimensions X, Y, Z).


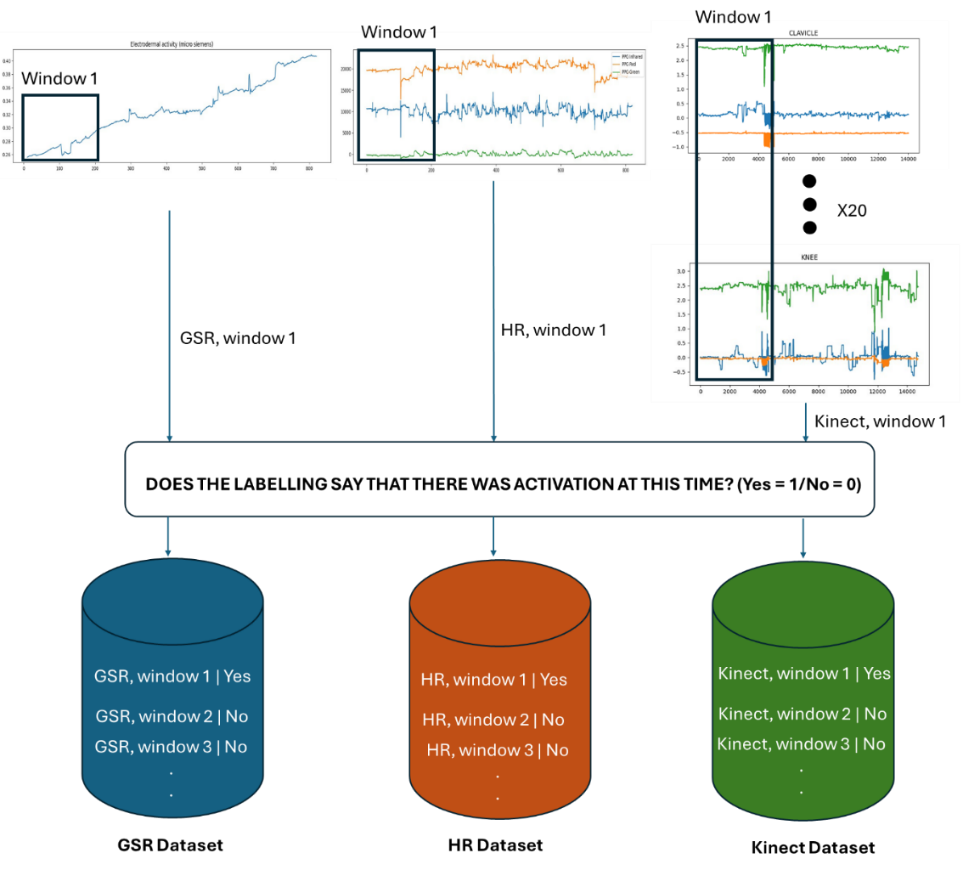


**Supplementary Figure 2.** Diagram illustrating the process from user signals to labeled data.

## Model Training

Once the dataset was constructed, the next step was to develop a model that could learn from it. However, the data stored in the dataset consists of raw time series, which poses a challenge: few ML models are able to handle raw time series directly for classification tasks. Therefore, the first step was to transform these sequences into fixed-length feature vectors—a process commonly referred to as feature extraction.

In traditional physiological signal processing, feature extraction from signals such as GSR may involve computing hand-crafted features, such as the number of phasic peaks or the average tonic level, over a window. However, this approach is computationally inefficient, difficult to scale, and impractical for real-time deployment, which is essential for the present use case.

To address this, a convolution-based approach was adopted. A convolution applies a kernel (i.e., a sequence of numerical weights) that is repeatedly multiplied with overlapping segments of the signal to extract temporal patterns. For example, if a time window contains 225 samples (15 seconds at 15 Hz), a kernel of size 7 is multiplied with the first 7 values, then slid across the signal—step by step—producing a new set of values that encode features of the original time series.

This operation can be seen as a form of automatic feature extraction, in contrast to manually designing features. For instance, if one wanted to extract the number of peaks in the phasic component of GSR, one could theoretically design a set of convolutional kernels to identify such patterns using signal processing theory.

Rather than engineering such kernels manually, a ROCKET (RandOm Convolutional KErnel Transform; Supplementary Figure 3) was employed, a state-of-the-art method for time-series classification. ROCKET automatically generates a large number of random convolutional kernels, effectively transforming each raw signal into thousands of informative features in a computationally efficient and scalable manner. This approach enabled the creation of a rich and diverse feature space without compromising the model's ability to function in real-time.

**
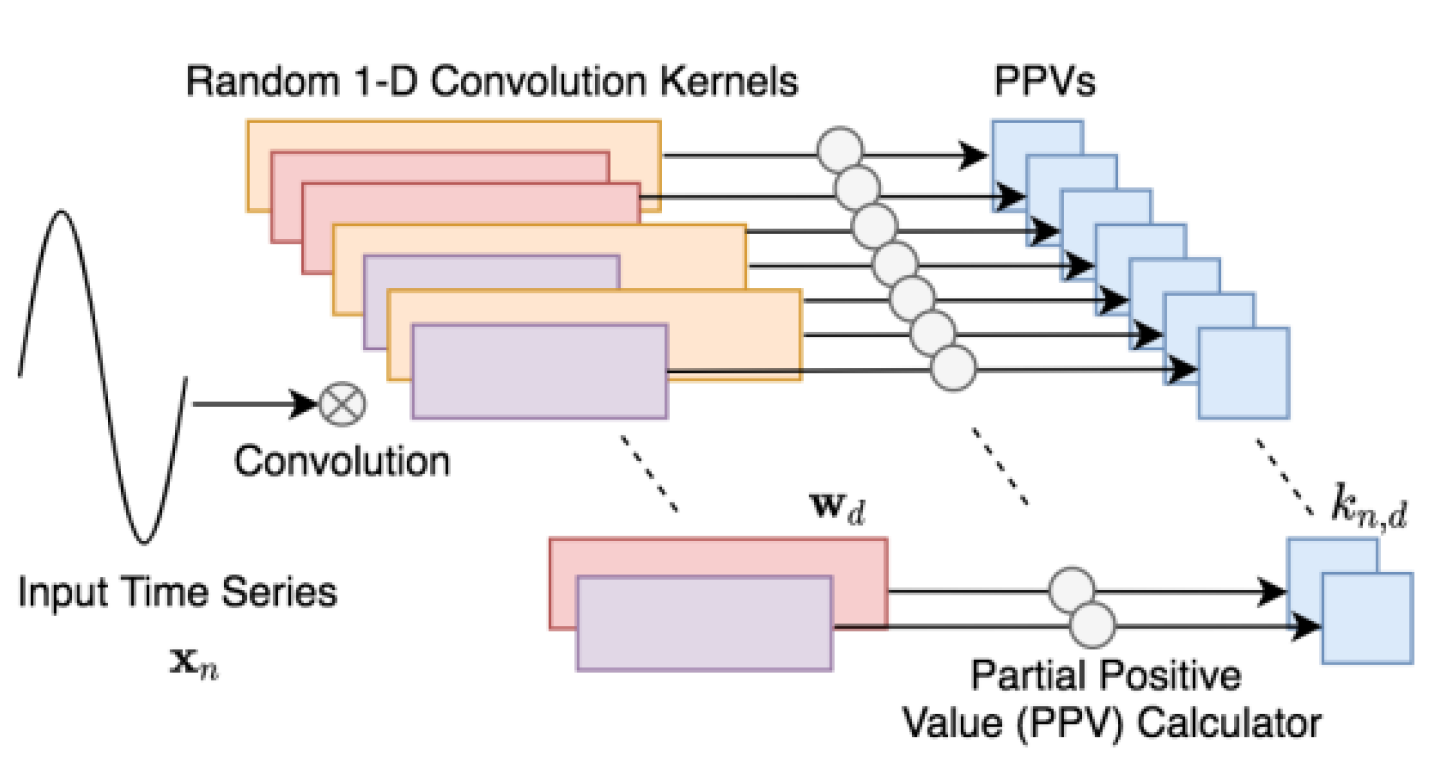
**

**Supplementary Figure 3.** ROCKET system.

Once the ROCKET model generates automatic features from the input signals, a final prediction must be made based on these features. To accomplish this, a model capable of learning effectively even when presented with a large number of features was employed: the Ridge Classifier. This classifier was trained to predict whether the child is in an activated or not-activated state, based on the ROCKET-derived feature vectors.

A visual representation of the full pipeline, from raw dataset to final model prediction, is shown in Supplementary Figure 4.


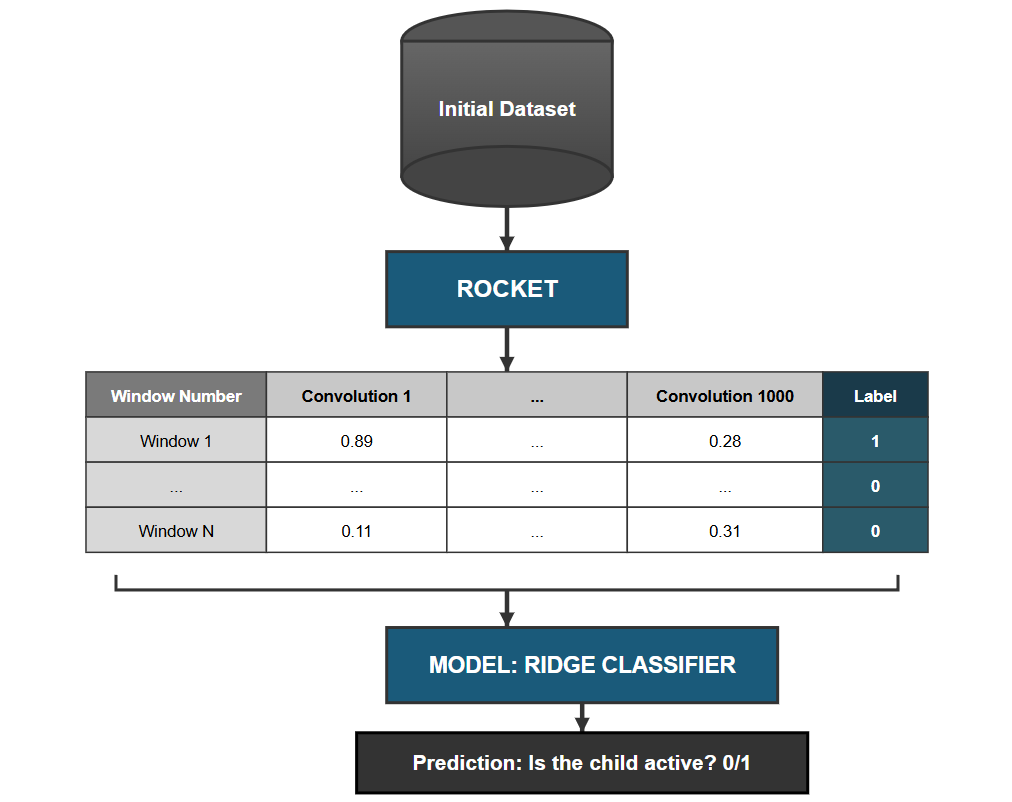


**Supplementary Figure 4.** Model explanation.

## Real-Time Inference Strategy

Although the offline-trained model demonstrated good performance, real-time inference presented several challenges, particularly due to class imbalance and the inherently noisy nature of biosignals. These issues were exacerbated by the need for immediate and reliable detection to support clinical applications. To address this, a robust decision-making strategy was developed and deployed in production.

The model operates in real time according to the following steps:

1. Signal Acquisition and Windowing: For each of the three biosignals (GSR, HR, and kinematic data), a 15-second sliding window is used to capture real-time input.
2. Feature Extraction: ROCKET transforms each window into a high-dimensional feature vector using the same kernel bank as in training.
3. Prediction: The trained Ridge Classifier processes each feature vector and returns a prediction: activated or not activated.
4. Temporal Smoothing: To improve prediction robustness, this process is repeated three times consecutively for each signal (i.e., every 5 seconds, a new 15-second window is evaluated). This results in:
   - 3 predictions per signal if only one modality is active,
   - 6 predictions if two modalities are active,
   - 9 predictions if all three signals are available.
5. Decision Rule:
   - If all three biosignals are available, the system triggers an AP if ≥3 of 9 predictions are activated.
   - If only one or two biosignals are available, ≥2 activated predictions (out of 3 or 6, respectively) suffice to trigger an adaptation.

This ensemble-like approach, using a majority-voting mechanism across time and modalities, enhances reliability and reduces the likelihood of spurious activations or false negatives caused by transient signal artifacts.

## Model Validation

The final model was validated using a held-out set of participants from the baseline trial who were not involved in training. A nested cross-validation protocol was applied to estimate generalizability and optimize hyperparameters without data leakage. The validation procedure included:

Defining Test Sets: Specific participants were reserved exclusively for testing, consistent across all signal modalities.

Model Training: Separate ROCKET + Ridge Classifier models were trained for each biosignal type, using 15-second windowed data.

Real-Time Inference Simulation: The inference process described above was applied to each test set, generating predictions for each modality and a final aggregated prediction using the voting system.

Performance Metrics: Accuracy, precision, recall, and F1-score were calculated to evaluate each model individually and the ensemble voting system as a whole.

Although single-modality models offered valuable insights into signal-specific discriminability, the final production-ready system relies exclusively on the aggregated voting classifier, as it demonstrated the highest robustness and practical reliability during testing (see Supplementary Table 2).

**Supplementary Table 2.** Performance Metrics Across Signal Modalities and Final Voting System.

| **Model** | **Accuracy^1^** | **Precision^2^** | **TPR^3^** | **TNR^4^** | **F1 score^5^** |
| --- | --- | --- | --- | --- | --- |
| Galvanic Skin Response | 95.3% | 2.3% | 3.1% | 98.0% | 0.026 |
| Heart Rate | 93.0% | 3.0% | 4.9% | 95.8% | 0.036 |
| Kinematic Data | 97.7% | 4.1% | 1.6% | 99.4% | 0.022 |
| **Voting Sistem** | **94.2%** | **5.0%** | **3.5%** | **97.7%** | **0.039** |

^1^Accuracy: percentage of correct classifications for activated/not activated.

^2^Precision: percentage of correct predictions among all instances predicted by the model as activated.

^3^True Positive Rate (TPR): percentage of actual activated instances correctly detected.

^4^True Negative Rate (TNR): percentage of actual not activated instances correctly detected.

^5^F1 score: a metric that evaluates the overall quality of the model based on the harmonic mean of TPR and Precision.

## Integration into the Virtual Reality Training Environment

To ensure usability and maintain user engagement, the adaptive ML model was integrated into the VR training protocol with predefined operational constraints. The objective was to balance system responsiveness with the need to avoid excessive interruption or user frustration.

The model triggers a brief AP outside the main VR task to help the child regulate psychophysiological arousal. The adaptation logic follows these rules:

1. One AP per Game Block: A maximum of six APs per session. Only the first detected activation in each block is acted upon; subsequent activations are suppressed to prevent excessive disruption.
2. Eligible Timing: APs can only be delivered immediately after specific game interactions (e.g., questions, repeated prompts). No AP is issued during ongoing task segments.
3. Temporal Decision Window: Activation decisions are based on biosignals captured during the 15 seconds preceding an eligible interaction moment.

The VR system autonomously receives input from the ML model and delivers APs in real time if the predefined activation thresholds are met.

### Implementation Challenges

Developing a real-time adaptive ML model in this context involved several methodological and practical challenges:

- Time-Series Modeling: Capturing informative patterns across heterogeneous time-dependent signals.
- Emotion Detection from Biosignals: Frustration and agitation do not manifest uniformly and can be subtle or masked.
- Class Imbalance: Very few high-arousal events relative to neutral states posed difficulties during training and validation.
- Indirect Labeling: Expert annotations rather than self-reports were used, increasing uncertainty in ground truth labels.
- Real-Time Constraints: Feature extraction and inference had to occur within strict latency windows using raw or minimally processed signals (e.g., avoiding full decomposition of GSR into phasic/tonic components).

Despite these constraints, the system demonstrated promising performance and feasibility for real-time deployment in experimental and clinical settings.

# Results

## Behavioral results

**Supplementary Table 3.** Significant within-session contrasts for acceleration metrics.

| Outcome | Effect tested | Statistic | *Adjusted p* (Tukey) | *EMM* | *SE* (EMM) | *SE* (Contrast) |
| --- | --- | --- | --- | --- | --- | --- |
| Accuracy | S1/S2 | *z*≈-0.711 | 0.8928 | NA(†) | NA | NA |
|  | S1/S3 | *z*≈-4.099 | 0.0002*** | S1= 0.777 S3= 0.869 | S1= 0.05 S3= 0.033 | 0.0827 |
|  | S1/S4 | *z*≈-4.351 | 0.0001*** | S1= 0.777 S4= 0.872 | S1= 0.05 S4= 0.032 | 0.0791 |
|  | S2/S3 | *z*≈-3.472 | 0.00293** | S2= 0.794 S3= 0.869 | S2= 0.047 S3= 0.033 | 0.0909 |
|  | S2/S4 | *z*≈-3.717 | 0.0012** | S2= 0.794 S4= 0.872 | S2= 0.047 S4= 0.032 | 0.0869 |
|  | S3/S4 | *z*≈-0.178 | 0.9980 | NA | NA | NA |
| Number of hints | S1/S2 | *z*≈-3.055 | 0.0121* | S1= 0.350 S2= 0.265 | S1= 0.087 S2= 0.066 | 0.120 |
|  | S1/S3 | *z*≈-6.551 | <0.0001*** | S1= 0.350 S3= 0.186 | S1= 0.087 S3= 0.046 | 0.1820 |
|  | S1/S4 | *z*≈-5.557 | <0.0001*** | S1= 0.350 S4= 0.210 | S1= 0.007 S4= 0.052 | 0.1540 |
|  | S2/S3 | *z*≈-3.628 | 0.0016** | NA(†) | NA | NA |
|  | S2/S4 | *z*≈-2.498 | 0.0602 | NA | NA | NA |
|  | S3/S4 | *z*≈-1.239 | 0.6019 | NA | NA | NA |
| (†)NA= not applicable | |  |  |  |  |  |

## Kinematic results

**Supplementary Table 4.** Significant within-session contrasts for acceleration metrics.

| Kinematic Mean Acceleration Statistics | | | | | | |
| --- | --- | --- | --- | --- | --- | --- |
| Outcome | **Effect tested** | **Statistic** | ***Adjusted p* (Tukey)** | ***EMM*** | ***SE* (EMM)** | ***SE* (Contrast)** |
| Head | S1/S2 | *z*≈-8.584 | <0.0001*** | S1= 0.163 S2= 0.20 | S1= 0.009 S2= 0.011 | 0.0196 |
|  | S1/S3 | *z*≈-11.256 | <0.0001*** | S1= 0.163 S3= 0.213 | S1= 0.009 S3= 0.012 | 0.0184 |
|  | S1/S4 | *z*≈-15.256 | <0.0001*** | S1= 0.163 S4= 0.235 | S1= 0.009 S4= 0.013 | 0.0167 |
|  | S2/S3 | *z*≈-2.677 | 0.0373* | S2= 0.20 S3= 0.213 | S2= 0.011 S3= 0.012 | 0.0226 |
|  | S2/S4 | *z*≈-6.713 | <0.0001*** | S2= 0.20 S4= 0.235 | S2= 0.011 S4= 0.013 | 0.0206 |
|  | S3/S4 | *z*≈-4.045 | 0.0003*** | S3= 0.213 S4= 0.235 | S3= 0.012 S4= 0.013 | 0.0219 |
| Body | S1/S2 | *z*≈-8.672 | <0.0001*** | S1= 0.126 S2= 0.160 | S1= 0.007 S2= 0.01 | 0.0218 |
|  | S1/S3 | *z*≈-10.811 | <0.0001*** | S1= 0.126 S3= 0.170 | S1= 0.009 S3= 0.01 | 0.0205 |
|  | S1/S4 | *z*≈-15.110 | <0.0001*** | S1= 0.126 S4= 0.191 | S1= 0.007 S4= 0.012 | 0.0182 |
|  | S2/S3 | *z*≈-2.144 | 0.1394 | NA(†) | NA | NA |
|  | S2/S4 | *z*≈-6.454 | <0.0001*** | S2= 0.160 S4= 0.191 | S2= 0.01 S4= 0.012 | 0.0232 |
|  | S3/S4 | *z*≈-4.322 | 0.0001*** | S3= 0.170 S4= 0.191 | S3= 0.01 S4= 0.012 | 0.0246 |
| Arm | S1/S2 | *z*≈-6.475 | <0.0001*** | S1= 0.320 S2= 0.378 | S1= 0.018 S2= 0.021 | 0.0219 |
|  | S1/S3 | *z*≈-8.961 | <0.0001*** | S1= 0.320 S3= 0.403 | S1= 0.018 S3= 0.023 | 0.0205 |
|  | S1/S4 | *z*≈-11.323 | <0.0001*** | S1= 0.320 S4= 0.429 | S1= 0.018 S4= 0.024 | 0.0193 |
|  | S2/S3 | *z*≈-2.494 | 0.0608 | NA | NA | NA |
|  | S2/S4 | *z*≈-4.850 | <0.0001*** | S2= 0.378 S4= 0.429 | S2= 0.021 S4= 0.024 | 0.0229 |
|  | S3/S4 | *z*≈-2.360 | 0.0850 | NA | NA | NA |
| Hand | S1/S2 | *z*≈-5.739 | <0.0001*** | S1= 0.466 S2= 0.539 | S1= 0.026 S2= 0.03 | 0.0218 |
|  | S1/S3 | *z*≈-8.714 | <0.0001*** | S1= 0.466 S3= 0.581 | S1= 0.026 S3= 0.032 | 0.0203 |
|  | S1/S4 | *z*≈-10.393 | <0.0001*** | S1= 0.466 S4= 0.606 | S1= 0.026 S4= 0.034 | 0.0194 |
|  | S2/S3 | *z*≈-2.983 | 0.0151* | S2= 0.539 S3= 0.581 | S2= 0.03 S3= 0.032 | 0.0234 |
|  | S2/S4 | *z*≈-4.652 | <0.0001*** | S2= 0.539 S4= 0.606 | S2= 0.03 S4= 0.034 | 0.0225 |
|  | S3/S4 | *z*≈-1.674 | 0.3375 | NA | NA | NA |
| Leg | S1/S2 | *z*≈-10.295 | <0.0001*** | S1= 0.116 S2= 0.164 | S1= 0.007 S2= 0.011 | 0.0240 |
|  | S1/S3 | *z*≈-12.469 | <0.0001*** | S1= 0.116 S3= 0.177 | S1= 0.007 S3= 0.012 | 0.0223 |
|  | S1/S4 | *z*≈-16.289 | <0.0001*** | S1= 0.116 S4= 0.202 | S1= 0.007 S4= 0.013 | 0.0196 |
|  | S2/S3 | *z*≈-2.183 | 0.1280 | NA | NA | NA |
|  | S2/S4 | *z*≈-6.034 | <0.0001*** | S2= 0.164 S4= 0.202 | S2= 0.011 S4= 0.013 | 0.0279 |
|  | S3/S4 | *z*≈-3.869 | 0.0006*** | S3= 0.177 S4= 0.202 | S3= 0.012 S4= 0.013 | 0.030 |
| Foot | S1/S2 | *z*≈-10.569 | <0.0001*** | S1= 0.123 S2= 0.180 | S1= 0.008 S2= 0.013 | 0.0246 |
|  | S1/S3 | *z*≈-12.613 | <0.0001*** | S1= 0.123 S3= 0.193 | S1= 0.008 S3= 0.014 | 0.0229 |
|  | S1/S4 | *z*≈-16.259 | <0.0001*** | S1= 0.123 S4= 0.221 | S1= 0.008 S4= 0.016 | 0.0201 |
|  | S2/S3 | *z*≈-2.059 | 0.1668 | NA | NA | NA |
|  | S2/S4 | *z*≈-5.732 | <0.0001*** | S2= 0.180 S4= 0.221 | S2= 0.013 S4= 0.016 | 0.0294 |
|  | S3/S4 | *z*≈3-.691 | 0.0013** | S3= 0.193 S4= 0.221 | S3= 0.014 S4= 0.016 | 0.0316 |
| (†)NA= not applicable | |  |  |  |  |  |

**Supplementary Table 5.** Significant within-session contrasts for displacement metrics.

| Kinematic Displacement Statistics | | | | | | |
| --- | --- | --- | --- | --- | --- | --- |
| Outcome | **Effect tested** | **Statistic** | **Adjusted *p* (Tukey)** | ***EMM*** | ***SE* (EMM)** | ***SE* (Contrast)** |
| Head | S1/S2 | *z*≈-9.538 | <0.0001*** | S1= 0.053 S2= 0.067 | S1= 0.003 S2= 0.003 | 0.019 |
|  | S1/S3 | *z*≈-11.749 | <0.0001*** | S1= 0.053 S3= 0.07 | S1= 0.003 S3= 0.004 | 0.0181 |
|  | S1/S4 | *z*≈-15.707 | <0.0001*** | S1= 0.053 S4= 0.077 | S1= 0.003 S4= 0.004 | 0.0165 |
|  | S2/S3 | *z*≈-2.213 | 0.119 | NA(†) | NA | NA |
|  | S2/S4 | *z*≈-6.193 | <0.0001*** | S2= 0.067 S4= 0.077 | S2= 0.003 S4= 0.004 | 0.0207 |
|  | S3/S4 | *z*≈-3.991 | 0.0004*** | S3= 0.07 S4= 0.077 | S3= 0.004 S4= 0.004 | 0.0218 |
| Body | S1/S2 | *z*≈-9.687 | <0.0001*** | S1= 0.042 S2= 0.055 | S1= 0.002 S2= 0.003 | 0.0207 |
|  | S1/S3 | *z*≈-11.597 | <0.0001*** | S1= 0.042 S3= 0.058 | S1= 0.002 S3= 0.003 | 0.0197 |
|  | S1/S4 | *z*≈-15.862 | <0.0001*** | S1= 0.042 S4= 0.065 | S1= 0.002 S4= 0.004 | 0.0176 |
|  | S2/S3 | *z*≈-1.912 | 0.2227 | NA | NA | NA |
|  | S2/S4 | *z*≈-6.199 | <0.0001*** | S2= 0.055 S4= 0.065 | S2= 0.003 S4= 0.004 | 0.0228 |
|  | S3/S4 | *z*≈-4.300 | 0.0001*** | S3= 0.058 S4= 0.065 | S3= 0.003 S4= 0.004 | 0.0240 |
| Arm | S1/S2 | *z*≈-7.076 | <0.0001*** | S1= 0.094 S2= 0.111 | S1= 0.005 S2= 0.006 | 0.0199 |
|  | S1/S3 | *z*≈-9.633 | <0.0001*** | S1= 0.094 S3= 0.118 | S1= 0.005 S3= 0.006 | 0.0187 |
|  | S1/S4 | *z*≈-12.252 | <0.0001*** | S1= 0.094 S4= 0.126 | S1= 0.005 S4= 0.007 | 0.0176 |
|  | S2/S3 | *z*≈-2.564 | 0.0507* | S2= 0.111 S3= 0.118 | S2= 0.006 S3= 0.006 | 0.221 |
|  | S2/S4 | *z*≈-5.181 | <0.0001*** | S2= 0.111 S4= 0.126 | S2= 0.006 S4= 0.007 | 0.0208 |
|  | S3/S4 | *z*≈-2.625 | 0.0430* | S3= 0.118 S4= 0.126 | S3= 0.006 S4= 0.007 | 0.221 |
| Hand | S1/S2 | *z*≈-5.745 | <0.0001*** | S1= 0.131 S2= 0.150 | S1= 0.007 S2= 0.008 | 0.0201 |
|  | S1/S3 | *z*≈-8.794 | <0.0001*** | S1= 0.131 S3= 0.161 | S1= 0.007 S3= 0.008 | 0.0188 |
|  | S1/S4 | *z*≈-10.7 | <0.0001*** | S1= 0.131 S4= 0.168 | S1= 0.007 S4= 0.009 | 0.0180 |
|  | S2/S3 | *z*≈-3.054 | 0.0121* | S2= 0.150 S3= 0.161 | S2= 0.008 S3= 0.008 | 0.0214 |
|  | S2/S4 | *z*≈-4.954 | <0.0001*** | S2= 0.150 S4= 0.168 | S2= 0.008 S4= 0.009 | 0.0205 |
|  | S3/S4 | *z*≈-1.909 | 0.2243 | NA | NA | NA |
| Leg | S1/S2 | *z*≈-10.871 | <0.0001*** | S1= 0.033 S2= 0.047 | S1= 0.002 S2= 0.003 | 0.0225 |
|  | S1/S3 | *z*≈-13.039 | <0.0001*** | S1= 0.033 S3= 0.05 | S1= 0.002 S3= 0.003 | 0.021 |
|  | S1/S4 | *z*≈-16.739 | <0.0001*** | S1= 0.033 S4= 0.057 | S1= 0.002 S4= 0.004 | 0.0187 |
|  | S2/S3 | *z*≈-2.172 | 0.131 | NA | NA | NA |
|  | S2/S4 | *z*≈-5.9 | <0.0001*** | S2= 0.047 S4= 0.057 | S2= 0.003 S4= 0.004 | 0.0264 |
|  | S3/S4 | *z*≈-3.752 | 0.0010** | S3= 0.05 S4= 0.057 | S3= 0.003 S4= 0.004 | 0.0282 |
| Foot | S1/S2 | *z*≈-11.225 | <0.0001*** | S1= 0.032 S2= 0.047 | S1= 0.008 S2= 0.013 | 0.0229 |
|  | S1/S3 | *z*≈-13.096 | <0.0001*** | S1= 0.032 S3= 0.05 | S1= 0.008 S3= 0.014 | 0.0215 |
|  | S1/S4 | *z*≈-16.721 | <0.0001*** | S1= 0.032 S4= 0.056 | S1= 0.008 S4= 0.016 | 0.0191 |
|  | S2/S3 | *z*≈-1.876 | 0.2382 | NA | NA | NA |
|  | S2/S4 | *z*≈-5.526 | <0.0001*** | S2= 0.047 S4= 0.056 | S2= 0.013 S4= 0.016 | 0.0277 |
|  | S3/S4 | *z*≈-3.673 | 0.0014** | S3= 0.05 S4= 0.056 | S3= 0.014 S4= 0.016 | 0.0294 |
| (†)NA= not applicable | |  |  |  |  |  |

# References

Barrett, L. F., and Russell, J. A. (1999). The structure of current affect: Controversies and emerging consensus. *Curr. Dir. Psychol. Sci.* 8(1), 10-14.

Maddalon, L., Altozano, A., Minissi, M. E., Marín-Morales, J., Lovaton Romero, G., Parsons, T. D., et al. (submitted manuscript). Adaptive VR intervention on social-cognitive skills in children with ASD: A feasibility study. Manuscript in review for publication in *Int. J. Clin. Health Psychol.*

Russell, J. A., and Barrett, L. F. (1999). Core affect, prototypical emotional episodes, and other things called emotion: dissecting the elephant. *J. Pers. Soc. Psychol.* 76(5), 805.
